# Experimental and computational investigation of the kinetic evolution of the glutaminolysis pathway and its interplay with the glycolysis pathway

**DOI:** 10.1002/2211-5463.13841

**Published:** 2024-06-12

**Authors:** Zohreh Mirveis, Nitin Patil, Hugh J. Byrne

**Affiliations:** ^1^ FOCAS Research Institute Technological University Dublin Ireland; ^2^ School of Physics and Optometric & Clinical Sciences Technological University Dublin Ireland

**Keywords:** glutaminolysis, glycolysis, metabolic pathways, numerical kinetic model, real‐time and *in situ* monitoring

## Abstract

Exploring cellular responses necessitates studying real‐time metabolic pathway kinetics, considering the adaptable nature of cells. Glycolysis and glutaminolysis are interconnected pathways fundamental to driving cellular metabolism, generating both energy and essential biosynthetic molecules. While prior studies explored glycolysis tracking, this research focuses on monitoring the kinetics of the glutaminolysis pathway by evaluating the effect of glutamine availability on glycolytic kinetics and by investigating the impact of a stimulator (oligomycin) and inhibitor (2DG) on the glycolytic flux in the presence of glutamine. Additionally, we adapted a rate equation model to provide improved understanding of the pathway kinetics. The experimental and simulated results indicate a significant reduction in extracellular lactate production in the presence of glutamine, reflecting a shift from glycolysis towards oxidative phosphorylation, due to the additional contribution of glutamine to energy production through the ETC (electron transport chain), reducing the glycolytic load. Oligomycin, an ETC inhibitor, increases lactate production to the original glycolytic level, despite the presence of glutamine. Nevertheless, its mechanism is influenced by the presence of glutamine, as predicted by the model. Conversely, 2DG notably reduces lactate production, affirming its glycolytic origin. The gradual increase in lactate production under the influence of 2DG implies increased activation of glutaminolysis as an alternative energy source. The model also simulates the varying metabolic responses under varying carbon/modulator concentrations. In conclusion, the kinetic model described here contributes to the understanding of changes in intracellular metabolites and their interrelationships in a way which would be challenging to obtain solely through kinetic assays.

## Graphical abstract

This study explores the dynamic interplay between glycolysis and glutaminolysis, essential pathways for cellular energy and biosynthesis. By examining how glutamine availability affects glycolytic kinetics and metabolic flux, we illuminate the complex interactions that drive cellular metabolism.

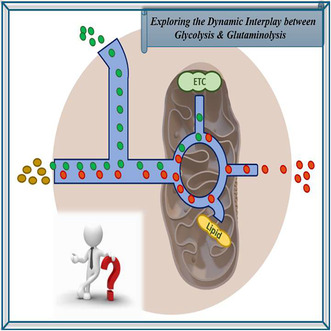

**Abbreviations**
2DG: 2‐deoxyglucose
Acetyl‐COA: acetyl coenzyme A
AMPK: AMP‐activated protein kinase
ATP: adenosine triphosphate
ECAR: extracellular acidification rate
ETC: electron transport chain
G‐6‐P: glucose‐6‐phosphate
GAP: glyceraldehyde 3‐phosphate
GDH: glutamate dehydrogenase
GLS: glutaminase
Glu: glutamate
Gly‐Model: glycolysis model
LDHA: lactate dehydrogenase A
Mal: malate
mTOR complex: mechanistic target of rapamycin complex
NAD+: nicotinamide adenine dinucleotide ions
NADH: nicotinamide adenine dinucleotide (reduced form)
NADPH: nicotinamide adenine dinucleotide phosphate
NH4+: ammonium ion
OAA: oxaloacetate
ODEs: ordinary differential equations
Oligo: oligomycin
PEP: phosphoenolpyruvate
Pyr: pyruvate
RFU: relative fluorescence units
Succ: succinate
TCA Cycle: tricarboxylic acid cycle
TCA: tricarboxylic acid
TRF: time resolved fluorescence
α‐KG: alpha‐ketoglutarate

Investigating cellular metabolism offers valuable insights into cellular functions, and their responses to both internal and external stimuli, by revealing changes in metabolic pathways [1]. The study of cellular metabolism has been advanced by two main approaches: metabolomics, which primarily focuses on identifying and quantifying metabolites within cells, and fluxomics, which examines the rates of metabolic reactions [2]. Fluxomics provides a kinetic representation of cellular phenotypes by revealing the kinetics of metabolic processes [3, 4]. To date, numerous studies have explored metabolic alterations in various diseases such as cancer [5], autoimmune disease [6], and neurodegenerative disorders [7] and have found a strong correlation between the kinetic changes of specific metabolic pathways, such as glycolysis and glutaminolysis, and the progression of these diseases [8]. Therefore, monitoring metabolic kinetics is essential for a better understanding of disease mechanisms and how cells respond or function in disorders, which is crucial for the development of effective therapeutic strategies [9].

In the realm of cellular metabolism, glycolysis and glutaminolysis serve as important pathways for energy production and biosynthesis of cellular building blocks [10]. Through the glycolysis mechanism, glucose is converted into pyruvate, which can either undergo oxidative phosphorylation in the mitochondria or be converted into lactate through fermentation in the cytosol. The path that pyruvate takes depends on energy demands and oxygen availability; while the glucose‐to‐lactate pathway is generally faster, it produces less energy compared to oxidative phosphorylation [11]. On the other hand, the glutaminolysis mechanism involves the conversion of glutamine into α‐ketoglutarate (α‐KG), which then goes through the tricarboxylic acid (TCA) cycle, replenishing its intermediates and ultimately leading to the production of pyruvate and subsequently lactate [12]. Recently, the glutaminolysis pathway has attracted attention for its key role in rapidly proliferating cells, serving as an alternative energy source to glycolysis in certain forms of cancer, a phenomenon known as “glutamine‐ addicted cancers” [13, 14].

Clearly, the presence of glutamine potentially influences the rate of the glycolytic pathway by fuelling the TCA cycle, which in turn draws more pyruvate into the mitochondria, facilitating the processing of pyruvate generated from glycolysis. Given this interplay, along with evidence that shows an upregulation of the kinetics of both pathways in various disorders [15, 16], recent fluxomic studies encourage the simultaneous monitoring of the kinetics of these two pathways [17, 18, 19]. Considering that cells often adapt their metabolic rates and switch between pathways such as glycolysis and glutaminolysis in response to local environmental conditions, the kinetic nature of these processes emphasises the necessity for real‐time and *in situ* monitoring. However, although real‐time and *in situ* monitoring techniques have been successfully employed for tracking the rate of the glycolysis pathway in living cells, monitoring the glutaminolysis pathway remains a challenge [20].

In this study, a kinetic assay (pH‐Xtra glycolysis assay) [21], designed for real‐time monitoring of glycolytic rates, was used to explore how glutamine influences these rates and to assess the impact of inhibiting and stimulating modulators on these pathways, and in turn how the assay, augmented by a phenomenological rate equation model, can be used to monitor the glutaminolysis process. The assay measures the extracellular acidification rate (ECAR) over time by tracking pH changes in the cell culture medium, which are correlated with lactate production. Although lactate is the primary source of extracellular acidification, CO_2_, a by‐product of mitochondrial respiration, also contributes [22]. Therefore, before the experiment, the cells were starved of glucose for 2 h to promote glycolysis over mitochondrial energy production.

While this assay provides valuable real‐time insights into the kinetics of glycolysis and glutaminolysis pathways and general metabolic shifts, it is limited to extracellular metabolic kinetics, and the data alone do not provide a comprehensive understanding of intracellular metabolic fluxes. As a result, a kinetic model, specifically designed to simulate the assay responses [23], was employed to obtain a deeper understanding of the changes in intracellular metabolite concentrations over time. Such a kinetic modelling approach also offers several advantages, including the ability to predict a pathway's behaviour under various conditions, identify potential drug targets such as key regulatory enzymes, and design experiments by predicting outcomes, thereby reducing both the cost and time required for experimental validation [23].

To summarise the aim of this study, as indicated in Fig. 1, an empirical investigation into the kinetics of the glutaminolysis pathway, and its impact on glycolytic rates, was first conducted using the pH‐Xtra assay, which measures the ECAR as a function of time. Subsequently, a kinetic model based on the experimental data was developed to further analyse the pathway and estimate metabolic fluxes, enabling potential responses to be simulated.

**Fig. 1:**
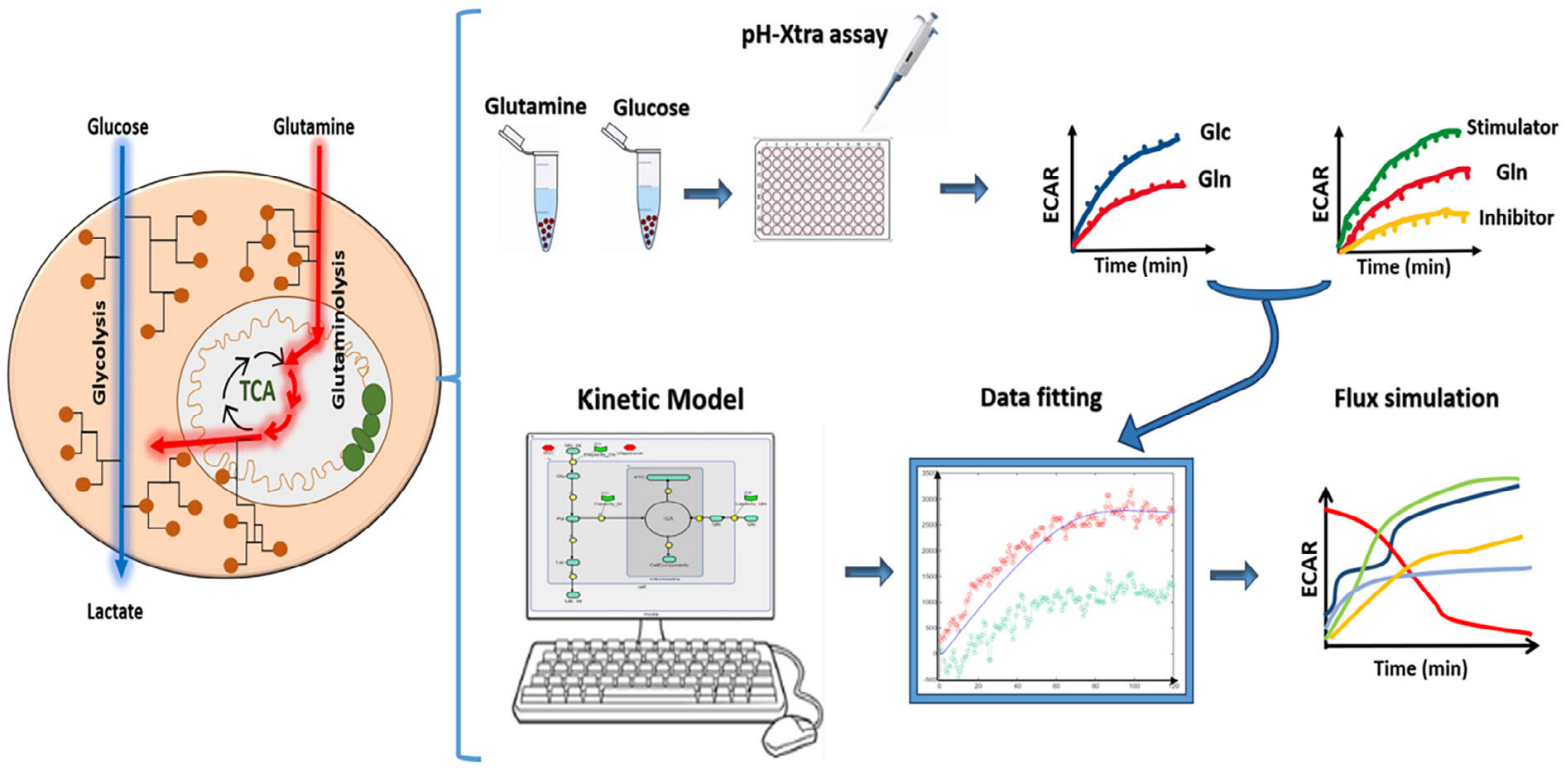
Depiction of the study objective: to empirically analyse the kinetics of glutaminolysis using the pH‐Xtra assay and to subsequently model the pathway to simulate metabolic fluxes.

## Glycolysis and glutaminolysis metabolic pathways

**Fig. 2:**
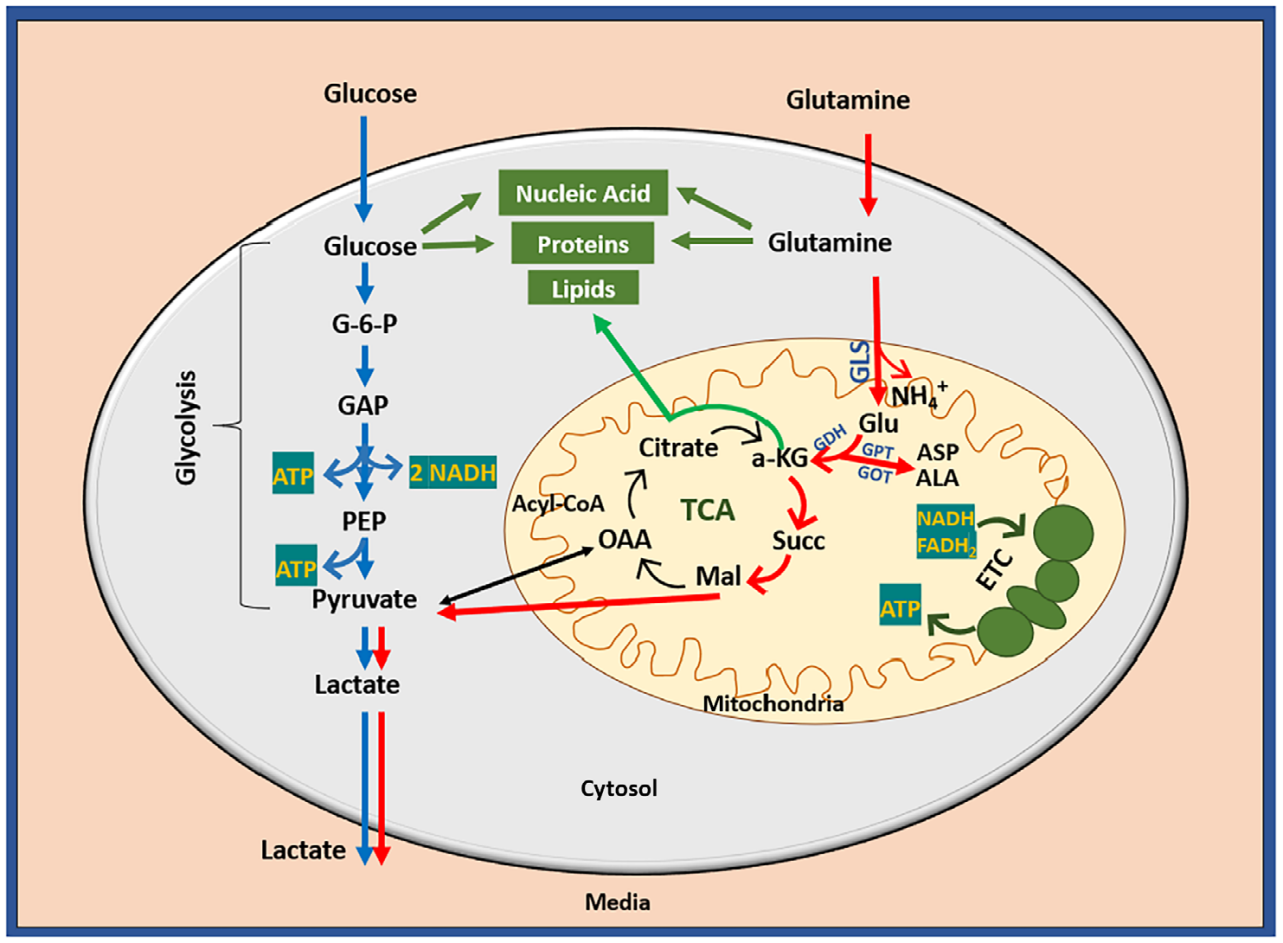
Glycolysis and Glutaminolysis metabolic pathway overview. Once internalised, glucose undergoes the glycolysis pathway which begins with a phosphorylation reaction, resulting in the formation of glucose‐6‐phosphate (G‐6‐P). This is followed by a series of three reactions leading to the production of Glyceraldehyde 3‐phosphate (GAP). GAP is then converted to phosphoenolpyruvate (PEP) through a series of four reactions, yielding ATP and NADH molecules. PEP is further metabolised to pyruvate, generating another ATP. Pyruvate can proceed to either oxidative phosphorylation in the mitochondria or lactate production in the cytosol, as indicated by the blue arrows. In parallel, glutamine follows the glutaminolysis pathway upon entering the mitochondria. Within the mitochondria, glutamine is converted to glutamate (Glu) by the enzyme glutaminase (GLS), with the generation of ammonia (NH₄^+^) as a by‐product. Glutamate can be further metabolised to α‐ketoglutarate (α‐KG) by glutamate dehydrogenase (GDH) or transaminases. Transaminases, including glutamate oxaloacetate transaminase (GOT) and glutamate pyruvate transaminase (GPT), also facilitate the synthesis of other amino acids such as aspartate (ASP) and alanine (ALA). Produced α‐KG can follow two pathways, in one pathway (indicated by the red arrows), α‐KG is oxidised through the TCA cycle, producing succinate (Succ) and malate (Mal), which exit the mitochondria and converts to pyruvate which subsequently generates lactate. In the alternative pathway (indicated by the green arrows), α‐KG is reduced to citrate via a reduction carboxylation process. Citrate exits the mitochondria and becomes involved in lipid production (Acetyl‐COA, Acetyl coenzyme A; OAA, Oxaloacetate).

Cells frequently switch their metabolic pathways to adapt to a range of bioenergetic and biosynthetic requirements [24]. This flexibility allows them to respond to varying needs for survival, growth, and longevity, while also adapting to nutrient availability and functional demands [6]. Among the complex network of metabolic pathways driving cellular functions, glycolysis and glutaminolysis are particularly important for cellular energy production and biosynthesis [25]. Figure 2 briefly illustrates the mechanisms of glucose and glutamine metabolism, as well as their synergy. Once glucose, the cell's primary energy source, is uptaken by a cell, it enters the glycolysis pathway, a series of enzyme‐catalysed reactions that result in pyruvate production (details of the pathway reactions are described in the caption of Fig. 2) [11, 26]. This pyruvate can either be converted to lactate under anaerobic conditions, yielding 2 Adenosine triphosphate (ATP) molecules (shown by blue arrows), or be directed to the mitochondria for oxidative phosphorylation under aerobic conditions [26, 27], potentially yielding up to 30–32 ATP molecules per glucose molecule [28]. While the aerobic pathway produces more ATP, it is slower than the anaerobic conversion of pyruvate to lactate, which swiftly regenerates Nicotinamide adenine dinucleotide ions, NAD^+^, for continued glycolysis. Consequently, under conditions of high energy demand or low oxygen availability, cells shift to the glycolytic pathway, that leads to lactate production, and this process is accelerated through the activation of signalling mechanisms like AMPK (AMP‐activated protein kinase) to maintain balanced ATP levels [29]. Additionally, glycolysis serves as a source of intermediates for various biosynthetic pathways, including (a) fuelling the pentose phosphate pathway for NADPH and ribose production, (b) creating glycogen from glucose 6‐phosphate, and (c) converting fructose 6‐phosphate for amino sugar synthesis in glycoproteins and glycolipids via the hexosamine biosynthetic pathway [30].

Similarly, glutamine is a key nutrient that performs multiple metabolic functions, including energy production by fuelling the TCA cycle, maintaining redox balance, and supporting the biosynthesis of nucleotides, proteins and lipids [20, 31]. Glutamine's primary metabolic role in bioenergetics starts upon its entry into the mitochondria, where it is initially deaminated to form glutamate in a reaction catalysed by glutaminase (GLS). This reaction also produces NH_4_^+^, a metabolic by‐product that triggers autophagy. Notably, the oncogene c‐Myc has been shown to induce GLS expression, thereby enhancing glutamine catabolism to support cell survival and proliferation [32]. Afterwards, glutamate can be oxidised to α‐ketoglutarate (α‐KG) by the enzyme glutamate dehydrogenase (GDH). GDH catalyses the reversible conversion between glutamate and α‐KG, using ammonium ions (NH₄^+^). This reaction can proceed in either direction, but it has been reported that, in the presence of high levels of ammonium, such as those produced during the catabolism of glutamine, GDH mainly converts α‐KG to glutamate [33]. Additionally, the conversion of glutamate to α‐KG can occur through a reversible transamination process facilitated by transaminases, including glutamate pyruvate transaminase (GPT, also known as alanine aminotransferase) and glutamate oxaloacetate transaminase (GOT, also known as aspartate aminotransferase). This process not only produces α‐KG but also generates nonessential amino acids such as aspartate and alanine [34]. This α‐KG then participates in the TCA cycle's oxidative process to produce the essential cofactors NADH and FADH_2_ that drive the electron transport chain (ETC) for energy generation [35]. Malate, derived from the TCA cycle, is converted into cytosolic pyruvate, which is then transformed into lactate by lactate dehydrogenase A (LDHA). This process, known as glutaminolysis and indicated by red arrows in Fig. 2, is upregulated in rapidly proliferating cells [33]. Also, α‐KG can enter the reductive carboxylation pathway, partially reversing the cycle to produce citrate, which leads to lipid synthesis. Interestingly, if the ETC is dysfunctional, some TCA cycle intermediates can still be produced through reductive carboxylation [36].

Both glycolysis and glutaminolysis are intricately linked pathways, particularly evident when α‐KG from upregulated glutaminolysis replenishes TCA cycle intermediates, thereby facilitating the processing of pyruvate generated from glycolysis. Additionally, both glucose and glutamine contribute to the biosynthesis of essential macromolecules such as nucleic acids, proteins and lipids [37]. Also, it has been reported that glutamine metabolism relies on the presence of glucose, which triggers glutamine uptake via the cytosolic hexosamine biosynthetic pathway which means cells cannot solely depend on glutamine as an alternative carbon source; sufficient glucose is required to sustain glutamine uptake [38].

Both pathways are notably upregulated in certain disorders, particularly cancers, influenced by a range of factors. For example, the mTOR complex responds to variables like nutrient availability and energy status, enhancing both glycolytic and glutaminolytic metabolic rates in high‐energy‐demanding condition like in cancer cells [39]. Inhibiting key enzymes in these pathways by some inhibitors currently in clinical trials has effectively slowed down cancer cell proliferation, emerging as a promising area of focus in cancer research [40]. Given the complex interplay between glycolysis and glutaminolysis and the promise of therapeutic interventions targeting them, recent fluxomics studies have begun to monitor both pathways in parallel [41].

## Materials and methods

### Experimental measurement

LLC‐MK2 cells (monkey kidney cells, generously donated by Prof. Ultan Power, Queen's University Belfast, UK) were cultured using Dulbecco's Modified Eagle Medium (DMEM; Sigma Aldrich, Wicklow, Ireland) enriched with 10% MSC‐Qualified Foetal Bovine Serum (FBS; Sigma Aldrich) and 1% penicillin–streptomycin (Penstrep; GIBCO, ThermoFisher, Dublin, Ireland), at a temperature of 37 °C in a humidified incubator with 5% CO_2_. The cells were seeded at a cell density of 6.5 × 10^4^ cells per square centimetre in a flat bottomed, white, 96‐well plate with 200 μL DMEM and incubated at 37 °C in a 5% CO_2_. After 16 h, when the cells were attached to the bottom of the plate, they were washed twice with glucose‐free respiration buffer (ingredients as per instructions provided with the assay: 70 mm NaCl, 1 mm K‐phosphate, 50 mm KCl, 2.4 mm CaCl_2_ and 0.8 mm MgSO_4_.) and incubated in 100 μL glucose‐free respiration buffer at 37 °C in a CO_2_ free incubator for 2 h. This incubation duration was recommended by the assay supplier [42], and serves to remove dissolved CO_2_ in the buffer and effectively starve the cells, prompting them to consume their carbon reserves.

To monitor the influence of glutamine on metabolism, the pH‐Xtra glycolysis assay was utilised, purchased from Agilent in Cork, Ireland. This assay includes a luminescent sensor reagent that allows direct and real‐time kinetic measurements of changes in extracellular acidification (pH) over a specified duration. Notably, the reagent is cell impermeable and is not consumed or quenched during the experimentation process [43]. After a 2‐h period of incubation with glucose‐free respiration buffer, the buffer was substituted with 90 μL of the prepared samples (detailed in the following paragraph) and 10 μL of pH‐Xtra reagent. The plate was immediately placed in the Tecan Genios microplate reader set to kinetic monitoring in single read Time Resolved Fluorescence (TRF) mode at Ex/Em 360/595 nm, temperature 37 °C, top read mode with 100 μs lag and 100 μs integration time and the gain value set to 176. The instrument was programmed for 350 cycles over 120 min (0.34 min per cycle).

Cells were exposed to one of four different sample types, namely (a) 7.5 mm glucose only (serving as a glycolysis pathway control), (b) 7.5 mm glucose plus 2 mm glutamine, (c) 7.5 mm glucose, 2 mm glutamine, and 2 μm Oligomycin (as a stimulator), and (d) 7.5 mm glucose, 2 mm glutamine, and 100 mm 2‐Deoxyglucose (2DG, as an inhibitor). The respiration buffer was used to maintain a constant total volume of 90 μL. Notably, the methods for determining the concentration values of glucose and glutamine are explained in the next “Experimental design” section.

The assay's response, as a measure of extracellular acidification, was monitored for up to 2 h for the LLC‐MK2 cell line, conducted in triplicate. The average values from these triplicates were used for further analysis. Data normalisation was carried out using a cell‐free signal control to eliminate any measurement drift over time. All experiments were performed in parallel on the same 96‐well plate to facilitate comparison. Note, although the TRF mode measures delayed fluorescence or phosphorescence, literature from the assay supplier and publications based on the assay commonly plot the assay response as Fluorescence, in Relative fluorescence units (RFU). This convention is adopted here.

### Experimental design

In investigating the kinetics of glutaminolysis, a logical approach is to exclusively supply glutamine to starved cells and observe the resultant changes in extracellular acidification (ECA) level. Thus, the experimental protocol was initiated by supplying varying concentrations of glutamine (2, 4, 6, 8 mm) to the cells. Additionally, a control group of cells was included, fed only with glucose (20 mm) to facilitate a more effective comparison. Figure S1a clearly illustrates that the various concentrations of glutamine did not significantly alter the ECA levels. This finding is in contrast to the significant rise in ECA induced by glucose, particularly notable in the first hour, as indicated by the blue curve. These observations could be attributed to the possibility that cells utilise glutamine for energy production or other biosynthetic processes, which do not result in lactate/acid production. Another potential explanation is the inability of cells to effectively internalise glutamine without the presence of glucose. This hypothesis is supported by a study demonstrating that glucose deprivation significantly reduces glutamine uptake, due to the essential role of glucose metabolism in the hexosamine biosynthetic pathway for glutamine uptake [38]. To verify the effectiveness of the pH‐Xtra assay, the cellular response to two different concentrations of glucose alone was also measured (20 and 7.5 mm); the results are presented in Fig. S1b. Therefore, it was decided to maintain a constant supply of glucose in further experiments to study the impact of glutamine availability as an additional carbon source.

The next step was to determine which of the two tested glucose concentrations would be most suitable to use. For this purpose, an experiment was conducted to compare cellular responses to two glucose concentrations (7.5 and 20 mm), both alone (as a control) and in combination with 2 mm glutamine. Given the prevalence of 2 and 4 mm glutamine in metabolomic research [44, 45] and the low physiological level of glutamine in plasma [46], we selected the lower concentration of 2 mm for our studies. The results are presented in Fig. S2. The ECA levels in response to 20 mm glucose showed no significant changes when combined with glutamine (Fig. S2a), which might be due to enzyme saturation. Conversely, a significant change in ECA was observed with 7.5 mm glucose when glutamine was added (Fig. S2b). Based on these findings, a 7.5 mm concentration of glucose was chosen as the consistent supply for all experiments. This serves as a control, allowing for comparative analysis of the results under different experimental conditions.

### Model development

The model used in this study is an expanded version of the Glycolysis model (Gly‐Model) originally developed by Patil *et al*. [23] to simulate the response of the glycolysis assay in three different cell lines. This model was constructed using symbiology version 6.3 in matlab (MathWorks, Natick, MA, USA), based on ordinary differential equations (ODEs). In this study, the core structure of the model was maintained, as well as the reaction equations and parameters, and adapted to include the glutaminolysis pathway.

**Fig. 3:**
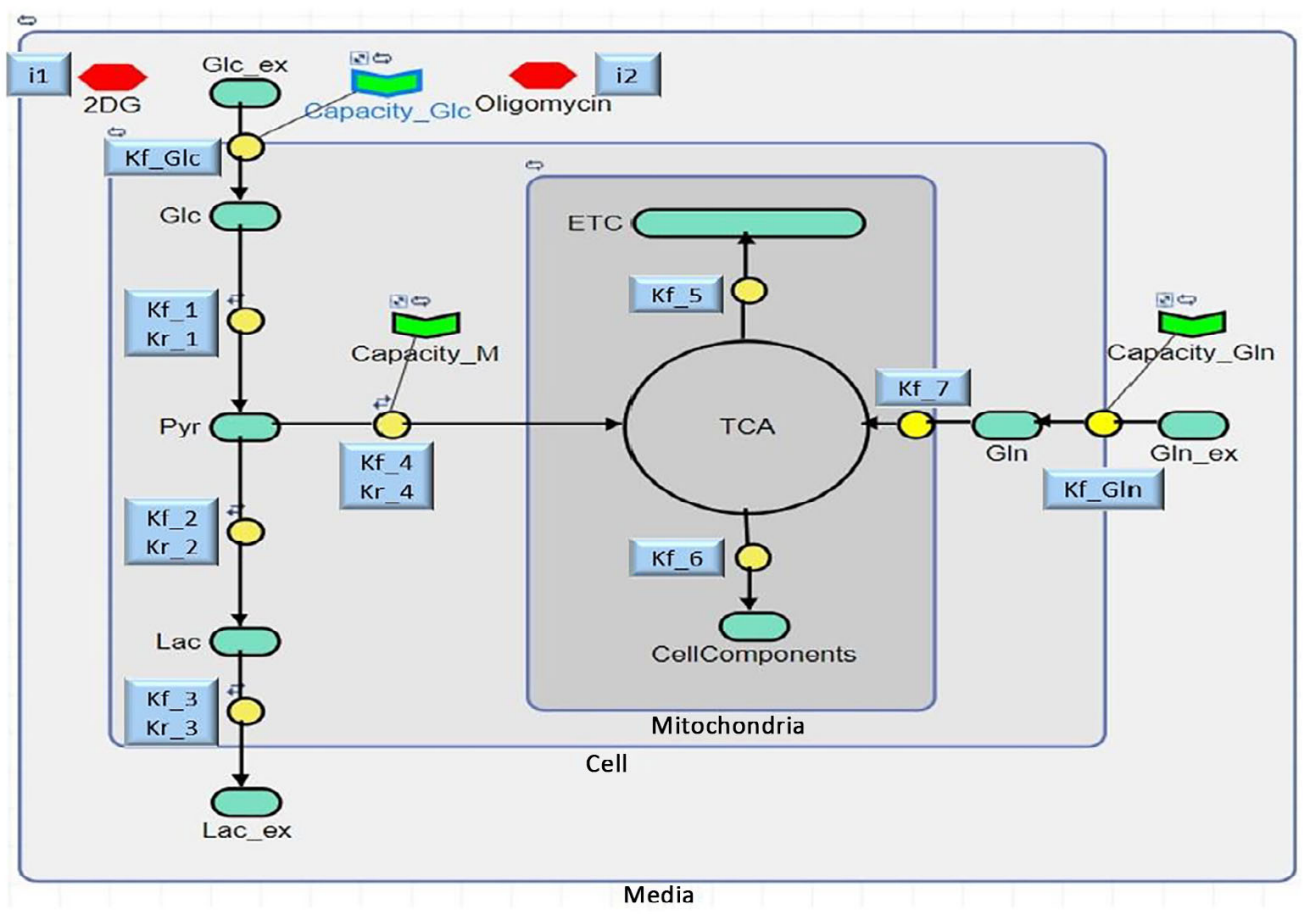
Kinetic Model of the assay response, including the Glycolysis and Glutaminolysis pathways: The model consists of 9 equations, including 4 reversible reactions, marked with a ‘⇋’ symbol. Components, shown in light green, are linked by yellow blocks as outlined in Table 1. These components are grouped into grey compartments based on their cellular location and the associated rate constants (‘k’) are displayed in blue boxes beside them. Drug modulators (2DG and Oligo) are red hexagons, and capacities are shown as green chevrons.

As shown in Fig. 3, the components of the model are compartmentalised, based on their cellular location. The largest compartment, labelled ‘medium’, contains reservoirs of glucose (Glu_ex) and glutamine (Gln_ex), which feed into the cell, as well as lactate (Lac_ex) produced by the cell. The ‘cell’ compartment covers the glycolysis pathway, by which internalised glucose (Glu) is converted to pyruvate (Pyr). This pyruvate either converts to lactate (Lac) or enters the smallest compartment, ‘mitochondria’. Additionally, the ‘cell’ compartment contains internalised glutamine, which also enters the ‘mitochondria’ compartment. Both pathways converge in the ‘mitochondria’ through the TCA cycle, which either fuels the ETC to produce energy or yields other by‐products such as lipids, proteins, etc. collectively termed ‘CellComponents’ in the model.

In the model, lines connecting two or more components via a reaction block (indicated by yellow circles) signify the transition of metabolites, each governed by a specific rate equation. These rate equations are based on ODEs (provided in Table S1) and generally follow the form “Reaction Rate = k * [Metabolite],” where “k” is the rate constant with units of per minute (min^−1^), and [Metabolite] represents the concentration of the metabolite involved, measured in micromolar (μm). Consequently, the overall Reaction Rate has the unit of μm·min^−1^. The specific rate equations used in the model vary and are each defined based on the type of reaction involved. For example, the rate of glucose uptake (Reaction 1) is equal to the constant rate of glucose uptake (kf_Glc) multiplied by the extracellular glucose concentration (Glu_ex), which is further multiplied by the glucose capacity (Capacity_Glc). This last term helps regulate the influx of glucose into the cell and serves as an overall representation of cellular glucose transport. A similar representation holds true for Capacity_Gln and Capacity_M, which refer to the capacities of glutamine uptake and mitochondrial function, respectively. Also, for reversible reactions such as Reaction 4, in which the rate constant k is defined by kf for the forward reaction and kr for the reverse reaction, the reverse rate equation is subtracted from the forward rate equation to represent the flow and direction of the metabolite. (If the rate equation value is positive, the forward reaction dominates; if negative, the reverse reaction does). This approach is consistently applied to other reactions, details of which are provided in Table 1. Moreover, in this study, the modulators 2DG and Oligomycin (Oligo) were used to investigate their effects on ECAR (primarily indicative of glycolytic flux) in the presence of glutamine, and the corresponding reaction equations were kept same as in the Gly‐Model. Notably, Patil *et al*. [23] have provided a detailed description of the mechanism of action for both modulators. 2DG, acting as an inhibitor with a rate of “i1,” and Oligo, acting as a stimulator with a rate of “i2,” were included in the rate equations at their respective sites of activity, following the form of ‘1 ± (modulator concentration * modulator rate)’, where ‘+’ is used for stimulators, resulting in an increase in flux rate, and ‘–’ is used for inhibitors, leading to a decrease in flux rate. The parameters i1 and i2 were introduced to represent the capacities of the cellular compartments to uptake the respective metabolites, and in other modelling approaches are represented as compartmental volume [47]. The values were determined empirically to provide the best performance of the model.

**Table 1:** List of reactions in the model and associated rate equations.

Sr. No.	Reaction	Rate equation
1	Glc_ex + Capacity_Glc → Glc	kf_Glc*Glc_ex*(Capacity_Glc)
2	Glc ↔ Pyr	kf_1* Glc*(1 + Oligomycin*i2‐[2DG]*i1)‐kr_1* Pyr
3	Pyr ↔ Lac	kf_2* Pyr ‐ kr_2* Lac
4	Lac ↔ Lac_ex	kf_3* Lac ‐ kr_3*Lac_ex
5	Pyr + Capacity_M ↔ TCA	kf_4*Pyr*(Capacity_M‐TCA)‐kr_4* TCA
6	TCA → ETC	kf_5*TCA*(1 + Gln/10)*(1‐Oligomycin*i2)
7	TCA → CellComponents	kf_6* TCA
8	Gln → TCA	kf_7*Gln
9	Gln_ex + Capacity_Gln → Gln	kf_Gln*Gln_ex*(Capacity_Gln)

In this section, an overview of the model's components, parameters, and rate equations has been provided. The methods for developing rate equations and the assignment of parameter values, based on fitting to experimental data, are discussed in detail in Section Kinetic model of glycolysis and glutaminolysis.

## Results and Discussion

### Monitoring system‐level kinetics of glycolysis & glutaminolysis pathway through a kinetic pH‐Xtra assay

**Fig. 4:**
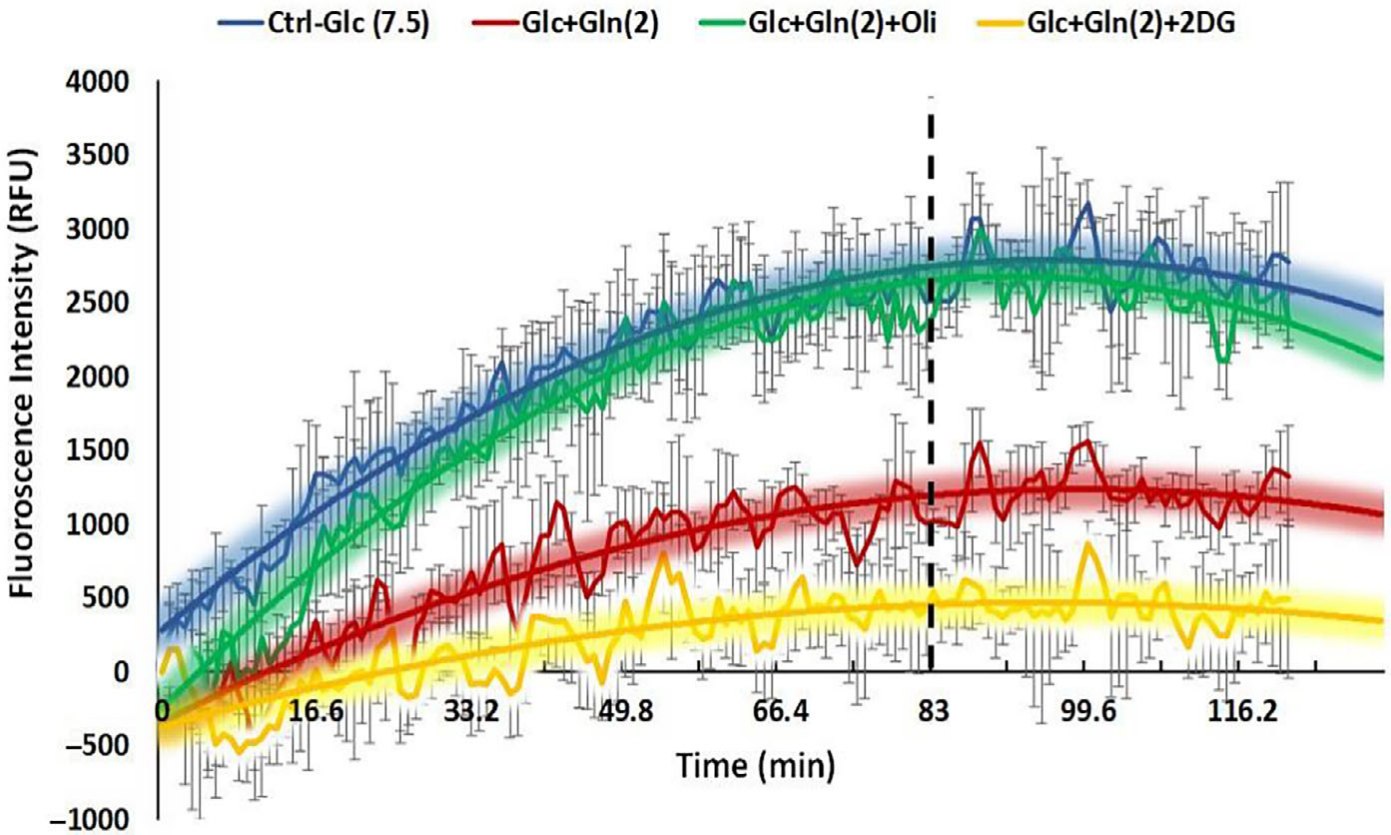
Time‐course assay response (up to 120 min) indicating extracellular acidification rate (ECAR) in LLC‐MK2 cell lines. The blue curve represents 7.5 mm glucose‐fed cells, red represents cells fed a mixture of 7.5 mm glucose and 2 mm glutamine. The green and yellow curves illustrate the effects of Oligomycin and 2DG additions to the glucose‐glutamine mixture, respectively. The error bars represent the standard deviation (STD) of triplicate measurements, and the dotted vertical line marks the beginning of plateau response phases observed after 83 min.

Considering the interplay between the glutaminolysis and glycolysis pathways and their shared end product (lactate), as discussed in Section Glycolysis and glutaminolysis metabolic pathways, this study employed the pH‐Xtra glycolysis assay to indirectly assess the kinetics of the glutaminolysis pathway by measuring the impact of glutamine presence on ECAR, which mostly results from glycolytic flux. This assay is specifically designed for real‐time monitoring of glycolytic rates and measures the ECAR by tracking pH changes in the cell culture medium which are attributable to lactate production, particularly in the case of glucose starvation. In the current experiments, the impact of glutamine on glycolytic flux was evaluated by measuring the ECAR in LLC‐MK2 cells exposed to either 7.5 mm glucose alone or a mixture of 7.5 mm glucose and 2 mm glutamine. The effects of modulators on this pathway were also assessed by adding the appropriate drug (2DG or Oligo) to the mixture of glucose and glutamine. Figure 4 shows the assay results for the LLC‐MK2 cell line, collected over a 120‐min period.

As the results indicate, when cells are fed solely with 7.5 mm glucose (shown by the blue curve), the ECAR increases until approximately the 83‐min mark, after which it begins to plateau, followed by a slight decrease in the latter stages. This expected increase in ECAR reflects heightened glycolytic activity introduced by cell starvation, surpassing oxidative phosphorylation and resulting in increased lactate production. The plateau observed is a result of approaching cellular steady state [48], and the subsequent decrease in ECAR, although not significant in this study, is more noticeable in the study conducted by Patil *et al*. [23], which monitored glycolysis over a longer period (140 min) and can be attributed to lactate reuptake by the cells for energy production purposes. This response acts as a control for glycolytic rate in the absence of glutamine. When both glucose (7.5 mm) and glutamine (2 mm) are present, a notable reduction in ECAR is observed (indicated by the red curve). Several factors can explain this trend. Firstly, the cells appear to be shifting their metabolic focus towards oxidative phosphorylation, which primarily generates ATP. As detailed in Section Glycolysis and glutaminolysis metabolic pathways, the presence of glutamine stimulates a more active TCA cycle, reducing the cell's reliance on glycolysis and, in turn, diminishing the lactate production. Secondly, the metabolism of glutamine produces ammonia, which may neutralise the lactate generated both intracellularly and extracellularly, helping to maintain pH balance [49]. Lastly, while glutamine itself may not directly influence LDH activity, some studies suggest that its derivative, α‐KG, might have the potential to decrease LDH functionality [50]. Notably, the cells were not fed with only glutamine because glucose is required to sustain the hexosamine pathway, which in turn supports the uptake of glutamine [38]. Therefore, all experiments used a constant concentration of 7.5 mm glucose to facilitate glutamine uptake.

Two modulators, with established effects on glucose metabolism, were also utilised to assess their impact on the ECAR in the presence of glutamine, as measured using the pH assay. The first modulator, Oligomycin, acts as a stimulator for ECAR by inhibiting the ATP synthase enzyme, thereby disrupting mitochondrial energy production via the ETC [29]. Adding Oligomycin to a mixture of glucose and glutamine, returned results similar to those observed with glucose alone (green plot in Fig. 4). This suggests that Oligomycin may counteract the reduction in ECAR caused by the effect of glutamine, consistent with the known mechanisms of both compounds: Oligomycin reduces mitochondrial metabolic activity, while glutamine increases its activity. The second modulator, 2DG, acts as a competitive inhibitor of the hexokinase and glucose 6 phosphate isomerase enzymes in the glycolysis pathway [51]. As shown by the yellow plot of Fig. 4, adding 2DG to a mixture of glucose and glutamine significantly reduced the ECAR in LLC‐MK2 cells. Given 2DG's mechanism of action, it is unlikely to affect the glutaminolysis pathway, which can also lead to lactate production. However, the lactate derived from glutamine is significantly less than that from glycolysis. Therefore, by inhibiting glycolysis, the overall ECAR response have been markedly reduced.

It is therefore demonstrated that, by employing this kinetic assay, valuable insights can be gained into the influence of glutamine on ECAR, thereby elucidating the glutaminolysis kinetics in real‐time and under *in situ* conditions. Nevertheless, this assay alone does not provide a comprehensive view of the intricate mechanisms of the metabolic network, as it primarily captures the system's overall kinetics and modulations. To better understand the underlying metabolic processes, the experimental assay was augmented with a kinetic model of the assay responses.

### Kinetic model of glycolysis and glutaminolysis

The kinetic model illustrated in Fig. 3 provides a simplified overview of cellular glycolysis and glutaminolysis processes, as measured using the commercial assay, aiming to simulate the kinetic changes in metabolites concentrations over a specific time. A key factor that determines the model's functionality is the rate of reactions which are mathematically formulated based on ODEs. For better comprehension, this section first discusses the development of rate equations, focusing primarily on the reactions involved in the glutaminolysis pathway, the expanded part of the model (the reactions in the glycolysis pathway have already been comprehensively described in the original model's paper [23], although they are briefly mentioned here). This section then proceeds to address the values of parameters used in these rate equations.

The glutaminolysis pathway begins with Reaction 9 in Table 1, developed as “kf_Gln * Gln_ex * (Capacity_Gln),” which is responsible for the entire process of glutamine uptake and is determined by three parameters. The first parameter, the constant rate of glutamine uptake (kf_Gln), refers to the steady rate at which glutamine is absorbed from the media into cells, and could be influenced by cell type, environmental conditions, and other variables [52]. The second parameter, Gln_ex, represents the amount of extracellular glutamine available to nourish the cells, and the third parameter, glutamine capacity (Capacity_Gln), controls cellular glutamine intake and reflects how cells interact with their environment based on their intrinsic capacity. This function of Capacity_Gln has been incorporated by setting a boundary condition on it, indicated by the “
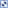
” symbol in the model (Fig. 3). The second stage in the glutaminolysis pathway involves the movement of intracellular glutamine into the mitochondria to participate in the TCA cycle. Although this stage consists of 2–3 distinct reactions, it is summarily represented in the model by Reaction 8, stated as “kf_7 * Gln.” In this equation, kf_7 stands for the constant rate of mitochondrial glutamine uptake, while Gln represents the intracellular concentration of glutamine. Furthermore, to reflect the role of glutamine in enhancing the activity of the TCA cycle and subsequently ETC for energy production, the term “(1 + Gln/10)” has been incorporated into the Reaction 6, which goes from the TCA to the ETC in the model. The term “Gln/10” accounts for the partial involvement of glutamine in glutaminolysis, as some intracellular glutamine contributes to cytosolic biosynthetic activities (like protein and nucleotide synthesis), and some glutamate derived from glutamine, exit the mitochondria before engaging the TCA cycle through the H+/Glutamate cotransporter [41].

To briefly describe the rate reactions for the glycolysis pathway (Reactions 1 to 7, as outlined in Table 1) as defined in the Gly‐Model [23]: Reaction 1 deals with cellular glucose uptake and employs the same equation used for glutamine uptake, although with different parameter values. The reversible Reaction 2 is representative of 10 enzymatic reactions of glycolysis pathway through which intracellular glucose “Glc” converts to pyruvate “pyr.” This reversible reaction abstracts the overall trends of both glycolysis and gluconeogenesis pathways, rather than directly representing each enzymatic reaction, some of which are indeed not reversible under physiological conditions. To account for the effects of the modulators Oligomycin and 2DG, the equation includes the term (1 + Oligomycin*i2‐[2DG]*i1). 2DG inhibits the first enzyme in the glycolysis pathway, while Oligomycin, despite inhibiting the ETC, indirectly stimulates glycolysis through cellular signalling mechanisms, primarily the AMPK signalling pathway [29], and thus is included as a representation of this stimulation. The product of Reaction 2, “pyr,” which is a key determinant in metabolic reprogramming, can follow two possible pathways. In the first, it undergoes two consecutive reversible Reactions 3 and 4, leading to the production of extracellular lactate (lac_ex), the corresponding equations for these reactions are represented simply by the basic form (K*[metabolite]) with appropriate parameters within Table 1. In the second pathway, it proceeds through another reversible reaction, 5, towards the mitochondria and ultimately enters the TCA cycle. Equation (5), in addition to its basic form, includes a multiplication term (Capacity_M‐TCA) in the forward reaction to indicate that the flux entering the mitochondria is regulated by the capacity of mitochondrial and TCA cycle. Reactions 6 and 7 are unidirectional and represent the end products of the TCA cycle: “ETC” and “Cell Components,” respectively. In the equation for Reaction 6, additional terms (1‐Oligomycin*i2) and (1 + Gln/10) are included to account for the effects of Oligomycin and glutamine, as previously discussed. The equation for Reaction 7 follows the basic form without additional terms.

Regarding the values of rate equation parameters, there are primarily two types of parameters that need to be determined in the model. The first is the rate constants (k, per hour), including both kf and kr, provided in Table S1. These rate constant values have been adjusted/optimised to closely fit the simulated lactate production of the model (green curve in Fig. 4) with the experimental ECAR data obtained from LLC‐MK2 cells (dotted navy blue curve in Fig. 4). Most of the rate constant values have been maintained the same as determined in the original Gly‐Model [23], marked by an asterisk (*) in the table. The only parameter that differs is Kr_3, which represents the constant rate of return reaction 3. In the current model, Kr_3 has been reduced to 0.01 h^−1^, compared to the Gly‐Model's value of 0.1 min^−1^. This difference indicates a significantly lower rate of lactate uptake by the cells compared to that in the Gly‐Model study, reflecting the variability of the assay.

In terms of nutrient uptake rates, the mechanisms for absorbing glucose and glutamine significantly impact their absorption rates by cells. Glucose is primarily absorbed through facilitated diffusion, a form of passive transport, allowing movement across the cell membrane along its concentration gradient, facilitated by specific transporters like those of the GLUT family [53]. On the other hand, glutamine, as an amino acid, often necessitates active transport coupled with Na^+^−dependent mechanisms for crossing the cell membrane through specialised transporters, particularly the SLC38 family [54]. Fourteen transporters in the mammalian cell plasma membrane mediate this process, detailed in the study of Bhutia *et al*. [54]. This active transport requires energy expenditure to move molecules against their concentration gradient. Consequently, it can be inferred that the uptake of glutamine through an active transport mechanism may be slower compared to glucose, due to the energy requirement and a more regulated process. Additionally, some studies have shown that glucose is consumed at a higher rate than glutamine. For example, one study on human umbilical endothelial cells (HUVECs) reported glucose and glutamine consumption rates of 8.54 and 2.32 nmol/10 000 cells·h^−1^, respectively [55]. Another study on the metabolic responses in baby hamster kidney cell cultures found maximum consumption rates of 32.5 nmol/(106 cells)/min for glucose and 6.5 nmol/(106 cells)/min for glutamine [56]. Hence, In the kinetic model, glutamine's lower consumption rate has been reflected by assigning it lower capacity and rate constant values compared to glucose, which is kept consistent with the Gly‐Model.

Besides the rate constants, the values of parameters acting as initial conditions for the model (provided in Table S3), such as Gln_ex, Glc_ex, and the capacities, need to be determined. Gln_ex and Glc_ex quantities are set to align with experimental conditions. Gln_ex is set to 2000 to represent the 2 mm glutamine concentration, and Glc_ex is set to 7500 to signify the 7.5 mm glucose concentration in the experimental media. In terms of the capacity values, considering that nutrient uptake capacity can vary based on factors such as cell type, metabolic state, and environmental conditions [38, 57], in the case of LLC‐MK2 cells, which are normal, healthy cells using glucose as the primary carbon source, the glucose uptake capacity is intentionally set higher (Capacity_Glc = 200) compared to that of glutamine (Capacity_Gln = 100). Also, the value of mitochondrial capacity is kept the same as what was determined in the Gly‐Model (Capacity_M = 50). The values of all other cellular components were set to zero under initial conditions. The capacities and compartments (media, cell, mitochondria, which are assigned a value of 1 in the Gly‐Model) are held constant (indicated by ‘
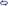
’ symbols above them), meaning they do not change over time, even when they are incorporated into the reaction.

### The model‐simulated responses to nutrient availability and modulator's impact

**Fig. 5:**
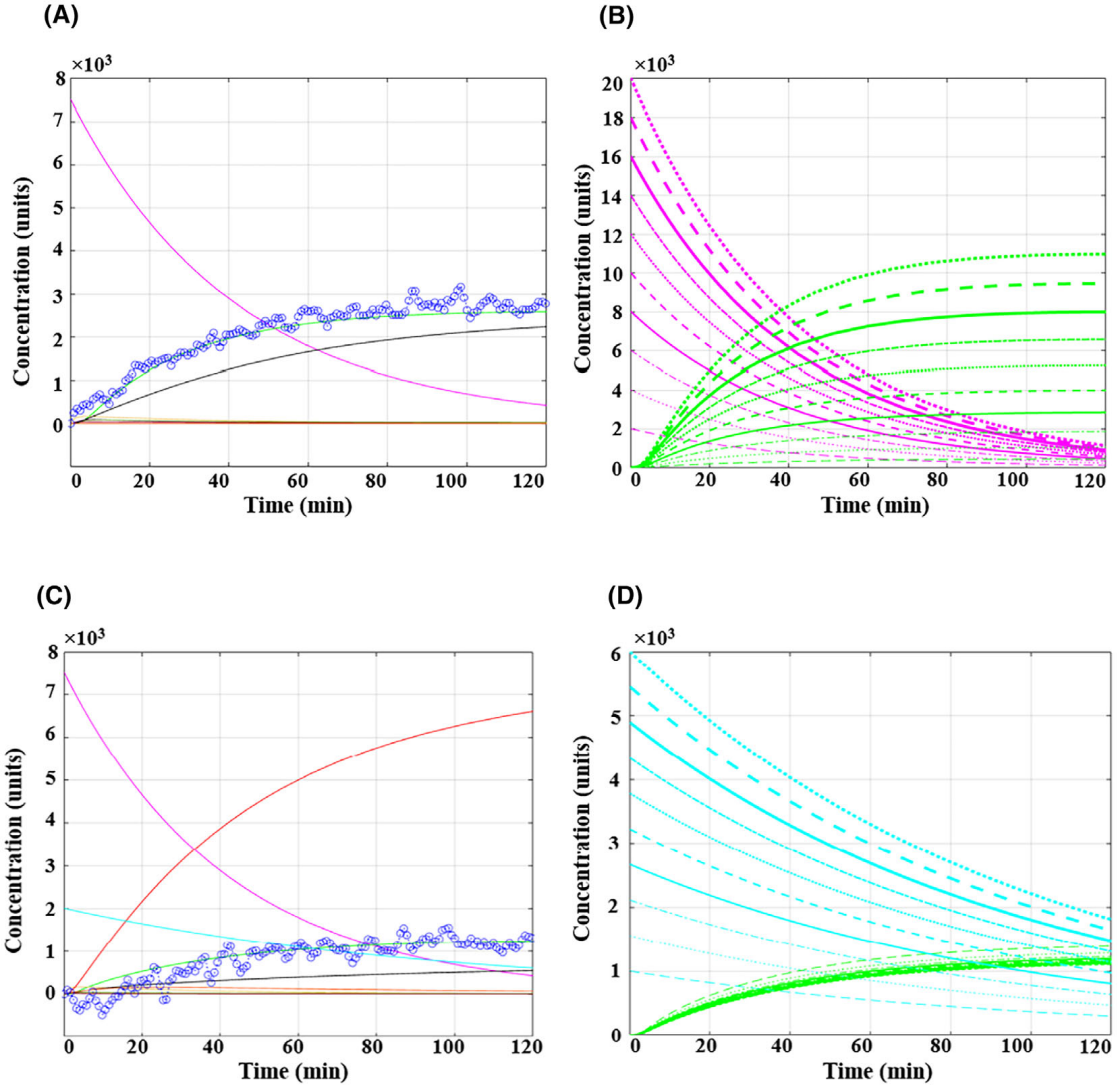
Model Simulation results under two different nutrient conditions, using the optimised parameters trained by overlaying simulated lactate production (the green curve) with the experimental ECAR data (dotted navy blue curve) from LLC‐MK2 cells. Different colours represent various metabolites in different compartments: media (Glc_ex in pink, Gln_ex in light blue, Lac_ex in green), cytosol (Glc in yellow, Pyr in purple, Lac in jade green, Gln in orange), and mitochondria (TCA in liver colour, ETC in red, Cell component in black). Figure part (A) and (B) depict the initial condition with only the glucose parameter (Glc_ex) set to 7500 units, while part (C) and (D) show both glutamine (2000 units) and glucose (7500 units) were set.

**Fig. 6:**
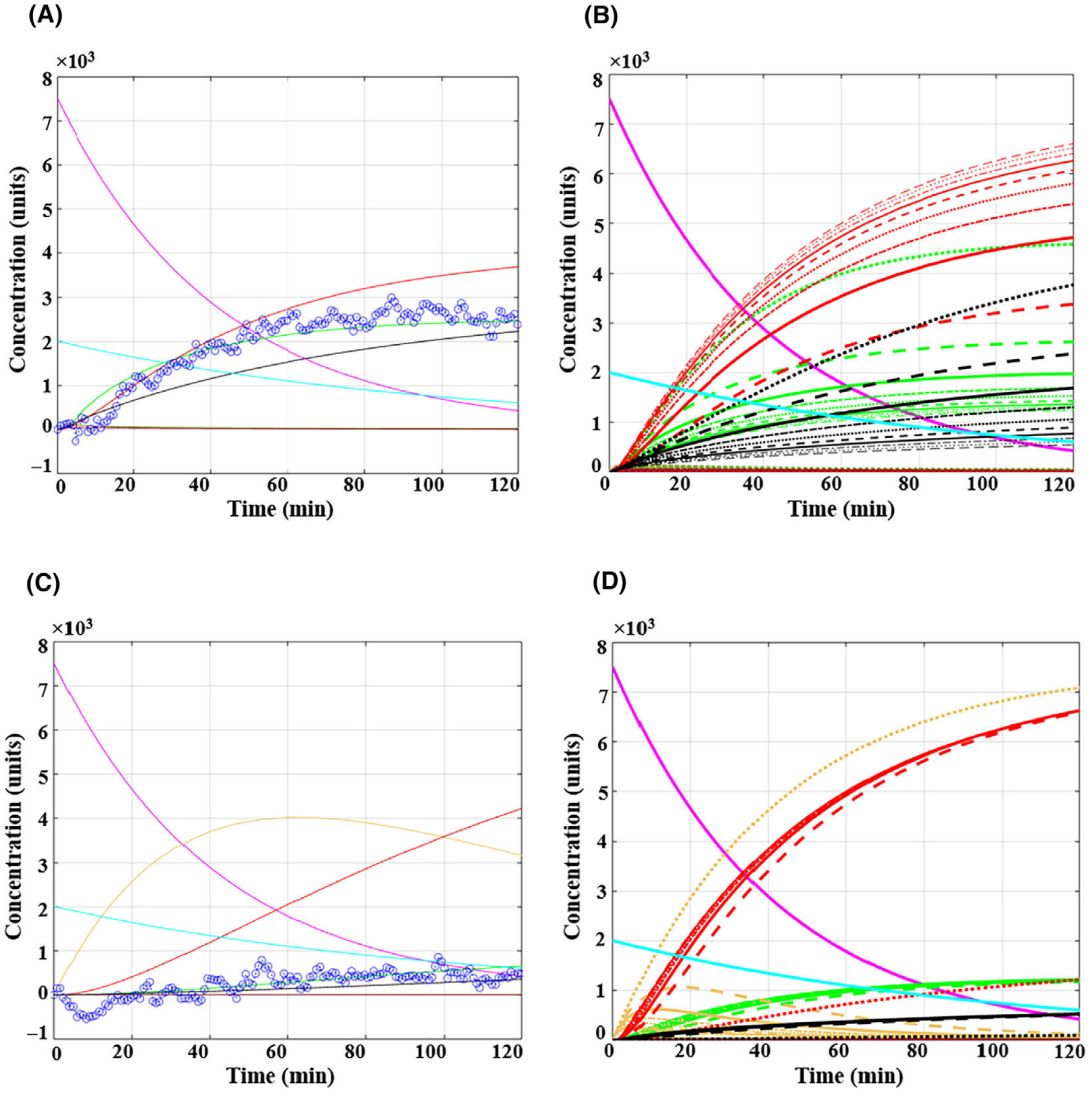
Model simulation results showing the effects of a stimulator (Oligomycin) and an inhibitor (2‐DG) on intracellular metabolite kinetics, using optimised parameters. Initial conditions for all simulations were glucose (Glc_ex at 7500 units) and glutamine (Gln_ex at 2000 units). Part (A) sets Oligomycin at 8.7 units for the best reproduction of the experimental data; part (B) varies Oligomycin from 1 to 10 units. Similarly, part (C) sets 2‐DG at 9.9 units for the best reproduction of the experimental data, and part (D) varies 2‐DG from 1 to 10 units. (Annotations: Glc_ex in pink, Gln_ex in light blue, Lac_ex in green, Glc in yellow, Pyr in purple, Lac in jade green, Gln in orange, TCA in liver colour, ETC in red, Cell component in black).

The model simulates and predicts changes in metabolite flux in response to different metabolic perturbations. Here, the model was employed to augment the commercially available assay, to better understand the influence of extracellular glutamine availability alongside glucose and how modulators (Oligo and 2DG) affect the kinetic responses of the cell. Figures 5 and 6 present the corresponding model simulation results, using optimised parameters trained on experimental ECAR data.

In Fig. 5, the model simulated two different initial conditions. In the first (A, B), cells were nourished with glucose alone, with Glc_ex set to 7500 units, and all other parameters set to zero. In the second (C, D), cells were fed both glucose (Glc_ex at 7500 units) and glutamine (Gln_ex at 2000 units). Assessing the simulated data in Fig. 5A, the pink curve is observed, representing glucose consumption and a simultaneous increase in the lactate green curve, which faithfully reproduces the experimentally observed responses (dotted navy blue curve) obtained from cells fed with only glucose (7.5 mm), confirming the optimised parameters. The red ETC and black cell component curves overlap (only the black curve is visible), and their increase is slower than that of lactate, suggesting a lower mitochondrial phosphorylation pathway activity compared to glycolysis. Due to the high glycolytic activity, certain intermediate metabolites further down the pathway, including intracellular glucose (yellow curve), pyruvate (purple curve), and lactate (jade green curve), undergo rapid changes, as once they are produced, they are quickly consumed by the subsequent reaction, making them indistinguishable at this level of observation. In Fig. 5B, the varying concentrations of glucose (ranging from 2000 to 20 000 units) and the associated produced lactate exhibit a direct relationship, as expected. This indicates that an increase in extracellular glucose concentration leads to a proportional rise in lactate production.

Figure 5C presents the simulated results from the second initial condition, in which, in addition to the glucose (pink curve), the consumption of glutamine is also observed (light blue curve). Compared to the previous condition (glucose alone), one notable difference introduced by the presence of glutamine is a significant decrease in the lactate production rate, nearly halving it. As discussed in section Kinetic model of glycolysis and glutaminolysis, this reduction is associated with the impact of glutamine on enhancing the activity of the TCA cycle and subsequently the ETC. This increase in activity draws more pyruvate into the mitochondria, leading to a decrease in lactate production. Additionally, the red ETC curve has significantly surpassed the lactate curve, indicating a metabolic shift from glycolytic to mitochondrial oxidative pathway. Similar to the first condition, intermediate metabolites rapidly change and their curves become unidentifiable. Moreover, as Fig. 5D illustrates, varying the concentration of glutamine (ranging from 1000 to 6000 units) has a negligible impact on lactate production. This finding is consistent with the assay's results, indicating that when the glycolysis pathway is blocked by 2DG, lactate production is mostly halted. However, lactate production gradually increases slightly over time, likely due to by‐products of the glutaminolysis pathway.

As discussed in section Monitoring system‐level kinetics of glycolysis & glutaminolysis pathway through a kinetic pH‐Xtra assay, the experimental data demonstrates that Oligomycin, when introduced to cells fed both glucose and glutamine, results in a substantial increase in ECAR. This increase closely resembles the ECAR levels observed in cells fed exclusively with glucose, suggesting that Oligomycin neutralised the reductive impact of glutamine on lactate production, in accordance with its known mechanism of action. However, the model's simulated data offers further insights into the impact of Oligomycin, shown in Fig. 6A. Although Oligomycin increases lactate production while decreasing the ETC rate compared to prior conditions (Fig. 5C), the ETC curve still surpasses the lactate curve, indicating the continued dominance of the oxidative phosphorylation pathway due to the influence of glutamine. Therefore, while the ECAR data for both conditions (glucose alone and Oligomycin impact) overlap, the model effectively distinguishes the underlying metabolic pathways.

Furthermore, the maintenance of the TCA cycle's activity by glutamine, coupled with Oligomycin's inhibition of one of its end products (ETC), results in an increase in the production of the other end product, “Cell components.” This effect is shown in Fig. 6B, whereby varying the concentration of Oligomycin from 1 to 10 units leads to a decrease in ETC production rates and a concurrent increase in production of “Cell components.” These components may include amino acids derived from anaplerotic intermediates (such as a‐KG and malate), or lipids, produced through a reductive pathway to compensate for energy requirements. Notably, even though cellular proliferation is negligible during the short incubation time of 2 h, the cells are inherently primed for growth and are actively setting the stage for subsequent proliferation, which the model aims to capture.

The model‐simulated results of 2DG's effect in Fig. 6C indicate an initial sharp drop in lactate production within the first hour, followed by a slight increase in the next hour. This is consistent with the experimental observations and can be attributed to 2DG's inhibition of the hexokinase enzyme in the glycolysis pathway, effectively blocking it. The small increase in lactate production might be a result of the increasingly activated glutaminolysis pathway, as supported by some studies. For instance, Haili *et al*. [58] reported that inhibiting glycolysis with 2‐DG stimulates glutamine metabolism, indicated by a dose‐dependent increase in β‐catenin and c‐Myc protein levels in DLD‐1 cells. Similar outcomes were observed in HepG2 and HeLa cells, suggesting that cells can adapt to glycolysis inhibition by activating the alternative glutaminolysis pathway [58]. Other metabolic analysis studies have also noted the activation of glutaminolysis as an alternative mechanism in response to glycolytic stress induced by the 2‐DG inhibitor [59]. Notably, the increase in ECAR may not solely originate from lactate because the conversion of glutamine into lactic acid involves the malic enzyme, which oxidatively decarboxylates malate, producing carbon dioxide, NADPH, and pyruvate [60]. Therefore, this increasing ECAR might be attributed to a combination of both lactic acid and CO_2_.

Moreover, the simulated data for 2DG demonstrates the accumulation of intracellular glucose (yellow curve) due to the blocked enzyme responsible for its consumption. The ETC curve is nearly half of what was observed in Fig. 5C, which is to be expected due to the interrupted transfer of pyruvate into the mitochondria. However, the ongoing increase in ETC confirms its sustained activity in the presence of glutamine. Additionally, examining the concentration variations of 2DG (ranging from 1 to 10 units), as depicted in Fig. 6D, reveals that, to a certain extent, alterations in its dosage do not markedly impact other metabolite changes. However, beyond a high dose threshold (~ 9 units), it exhibits a substantial inhibitory effect, contrary to Oligomycin, where each dose resulted in distinct alterations in metabolites. Notably, the associated simulated lactate curve for the high dose cannot be discerned due to overlap with a black curve.

## Conclusion

Simultaneously monitoring the glutaminolysis and glycolysis pathways is imperative due to their intricate interplay, influencing each other's flux adjustments for cellular adaptation. Although fluxomic techniques offer a deeper understanding of these pathways and valuable insights into their dynamic adjustments, real‐time analysis remains challenging due to its reliance on metabolomics, which is inherently destructive [20]. In this study, the first empirical phase employed the pH‐Xtra glycolysis assay, measuring ECAR, as representative of the lactate production rate, which could effectively assess the influence of the presence of glutamine in the medium on glycolytic flux and also evaluate the impact of the modulators on these pathways in LLC‐MK2 cells. In summary, the assay's results demonstrated that, when cells were supplied with glucose alone, the ECAR continuously increased up to approximately 80 min, plateaued and then started to decrease. This increase in ECAR indicates increased glycolytic activity, leading to increased lactate production. This data served as a control for glycolytic rate in the absence of glutamine, against which the influence of glutamine presence could be compared. When both glucose and glutamine were present, a significant reduction in ECAR was observed (almost halved), suggesting several factors might contribute to this trend such as, (a) a metabolic shift towards oxidative phosphorylation pathway due to glutamine's stimulation of a more active TCA cycle, reducing reliance on glycolysis, (b) the production of ammonia from glutamine catabolism which neutralises lactate to help maintain pH balance, and (c) α‐KG, a glutamine derivative, that may reduce LDH functionality, thereby reducing lactate production. Regarding the impact of the modulators on ECAR, in the presence of both nutrients, the assay's results demonstrated that the two modulators used in this study, Oligomycin as a stimulator and 2DG as an inhibitor, significantly altered the ECAR kinetics, as expected. Oligomycin increased ECAR to the level observed in the glucose alone experiment, implying that oligomycin neutralised the reductive effect of glutamine. On the other hand, 2DG notably reduced ECAR, indicating that lactate production primarily originates from the glycolytic pathway.

While the pH‐Xtra assay offers a cost‐effective option compared to high‐throughput omics methods for studying cellular metabolism kinetics *in vitro* and provides valuable insights into glycolysis, glutaminolysis, and overall metabolic changes, it does not, on its own, provide a comprehensive understanding of intracellular metabolic fluxes. Therefore, in the second phase of this study, an already validated kinetic model for the glycolysis assay (GLy‐Model) was employed and expanded to incorporate the glutaminolysis pathway. This model was trained using the experimental ECAR data obtained from the first phase and was used to simulate the changes in intracellular metabolite concentrations under different conditions (nutrients and modulators availability) over a 2‐h time frame. Consequently, it elucidated more detailed insights into metabolic kinetic responses. For instance, the model revealed how the availability of glutamine alongside glucose led to a metabolic shift from glycolytic to mitochondrial oxidative pathways, as evident by ETC curve surpassing the lactate curve, resulting in reduced lactate production. The model also illustrates the varying extracellular glutamine concentrations do not influence lactate generation. Furthermore, the model effectively differentiated the underlying pathway mechanisms behind two overlapping ECAR data sets obtained from two different conditions (glucose alone and Oligomycin impact experiments). Additionally, the model allows for the assessment of various concentrations of substrates and modulators, providing a more comprehensive understanding of how these factors affect metabolic kinetics. In summary, the kinetic model augments assay results by providing a more sophisticated understanding of changes in intracellular metabolites and their interrelationships. This level of insight would be challenging to obtain solely through kinetic assays.

However, there are some limitations to the model that should be considered. First, the model's simplifications involve simplifying complex metabolic processes into mathematical equations, may overlook important details and intricacies of cellular metabolism. Also, there are other significant pathways, such as the pentose phosphate pathway, that influence the kinetic changes but have not been considered in the model. Furthermore, the model's parameters were optimised based on the experimental data, potentially introducing biases or inaccuracies when the data is limited or subject to experimental errors. It is important to consider that cellular responses can vary depending on their type and condition since different cells have diverse metabolic profiles and responses. The model may not account for this variability, as this study's model validation used a specific cell type, LLC‐MK2, and a limited range of experimental conditions. Additionally, the model is limited in its ability to provide insights into the mechanisms by which glutamine influences other intracellular metabolites and their interrelationships, which are essential for altering glycolytic flux. This limitation restricts the ability to fully validate the model's effectiveness. In short, while the model offers valuable insights into potential changes in intracellular dynamics and interactions among various metabolites, it should be used cautiously, and its results should be interpreted within the context of its simplifications and potential biases. It is worth noting that, in this study, the model serves as a complementary approach, designed to provide preliminary predictions of metabolic changes, thereby guiding experimental design by suggesting potential outcomes and interactions. Its predictive nature is suited for hypothesising about complex biological systems and is not intended to offer definitive conclusions without experimental validation. Therefore, further validation and refinement may be necessary to enhance its accuracy and applicability to a broader range of conditions and cell types. Ideally, this would involve more precise techniques such as vibrational spectroscopy, which could be pursued in subsequent studies.

## Conflict of interest

The authors declare no conflict of interest.

## Author contributions

ZM: Conceptualisation, Writing – Original Draft, NP: Conceptualisation HJB: Conceptualisation, Writing – Original Draft, Supervision, Funding acquisition.

## Supporting information


**Fig. S1.** a) Cellular responses to ECA kinetic changes when exposed to various glutamine concentrations (2, 4, 6, 8 mM) compared to 20 mM glucose (blue plot), with glutamine response plots overlaid. b) Comparison of cellular responses (ECA levels) to glucose at concentrations of 7.5 mM (green plot) and 20 mM (blue plot).
**Fig. S2.** Cellular responses to ECA kinetic changes with glucose alone (blue plot) versus a mixture of glucose and 2 mM glutamine (red plot).
**Table S1.** List of ordinary differential equations which are the basis of the model.
**Table S2.** Optimised values of rate constants (μM·min^−1^) in the kinetic model.
**Table S3.** Optimised values of initial condition parameters in the kinetic model.

## Data Availability

The original model (Gly‐Model) can be accessed in the BioModels repository (model identifier: MODEL2312220001). This study also includes Supplementary information, comprising Tables S1 and S2.
