# Supplementary material for: Experimental and computational investigation of the kinetic evolution of the glutaminolysis pathway and its interplay with the glycolysis pathway
Source: FEBS Open Bio. 2024 Jun 12;14(8):1247–63. doi: 10.1002/2211-5463.13841 (PMC11301260; doi:10.1002/2211-5463.13841)
Supplement: Supplementary file 1 — Fig. S1. a) Cellular responses to ECA kinetic changes when exposed to various glutamine concentrations (2, 4, 6, 8 mM) compared to 20 mM glucose (blue plot), with glutamine response plots overlaid. b) Comparison of cellular responses (ECA levels) to glucose at concentrations of 7.5 mM (green plot) and 20 mM (blue plot). Fig. S2. Cellular responses to ECA kinetic changes with glucose alone (blue plot) versus a mixture of glucose and 2 mM glutamine (red plot). Table S1. List of ordinary differential equations which are the basis of the model. Table S2. Optimised values of rate constants (μM·min−1) in the kinetic model. Table S3. Optimised values of initial condition parameters in the kinetic model. [file FEB4-14-1247-s001.pdf]

Experimental and computational investigation of the kinetic evolution of the glutaminolysis pathway and its interplay with the glycolysis pathway

Zohreh Mirveis[1,2]*, Nitin Patil[1,2], Hugh J. Byrne[1]

[1]FOCAS Research Institute, Technological University Dublin, City Campus, Camden Row, Dublin 8, Ireland

[2]School of Physics and Optometric & Clinical Sciences, Technological University Dublin, City Campus, Grangegorman, Dublin 7, Ireland

**Corresponding Author:* D21127294@mytudublin.ie

Supplementary Material

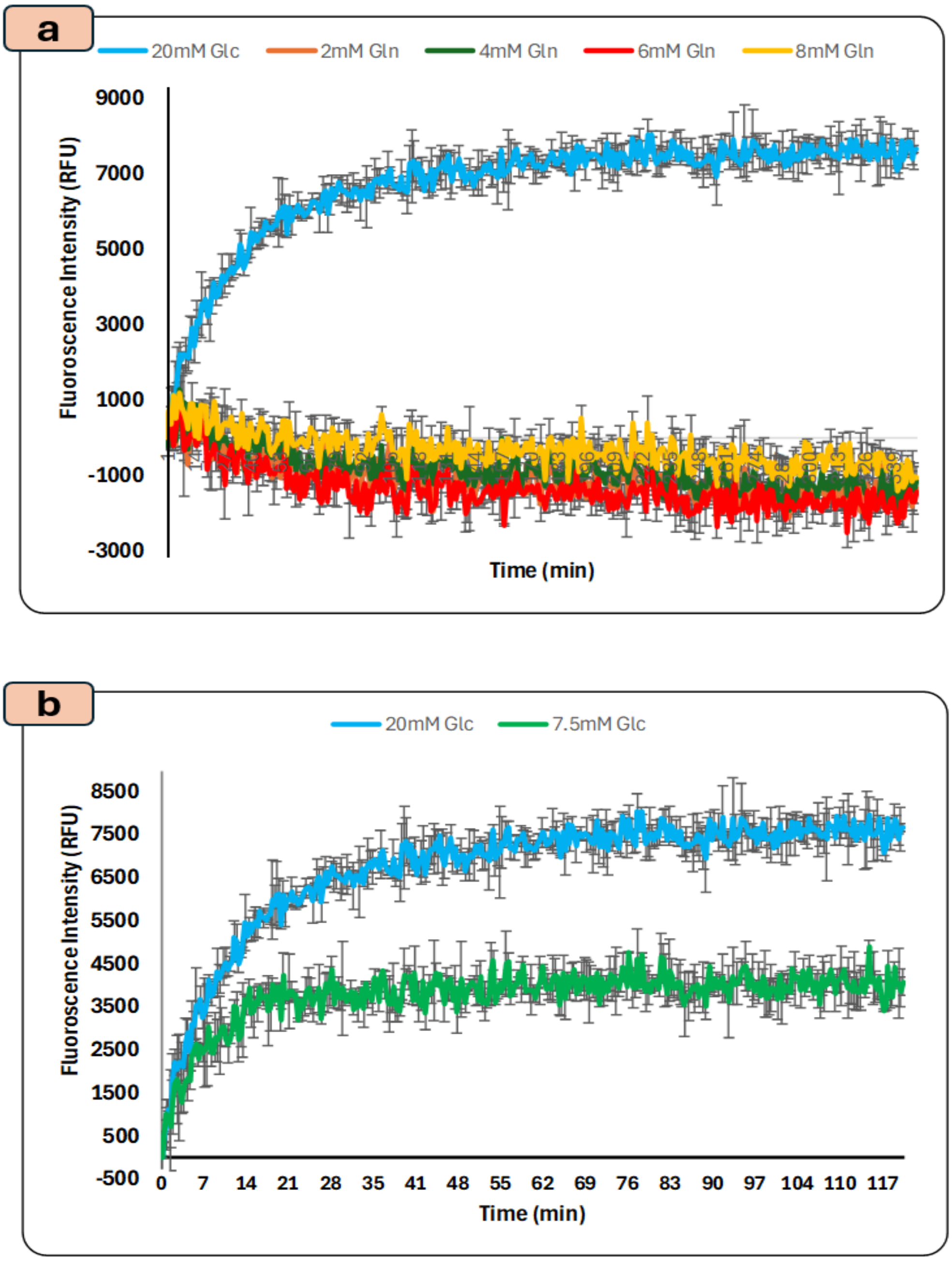

Figure 1S. a) Cellular responses to ECA kinetic changes when exposed to various glutamine concentrations (2, 4, 6, 8 mM) compared to 20 mM glucose (blue plot), with glutamine response plots overlaid. b) Comparison of cellular responses (ECA levels) to glucose at concentrations of 7.5 mM (green plot) and 20 mM (blue plot). The error bars represent the standard deviation (STD) of triplicate measurements.

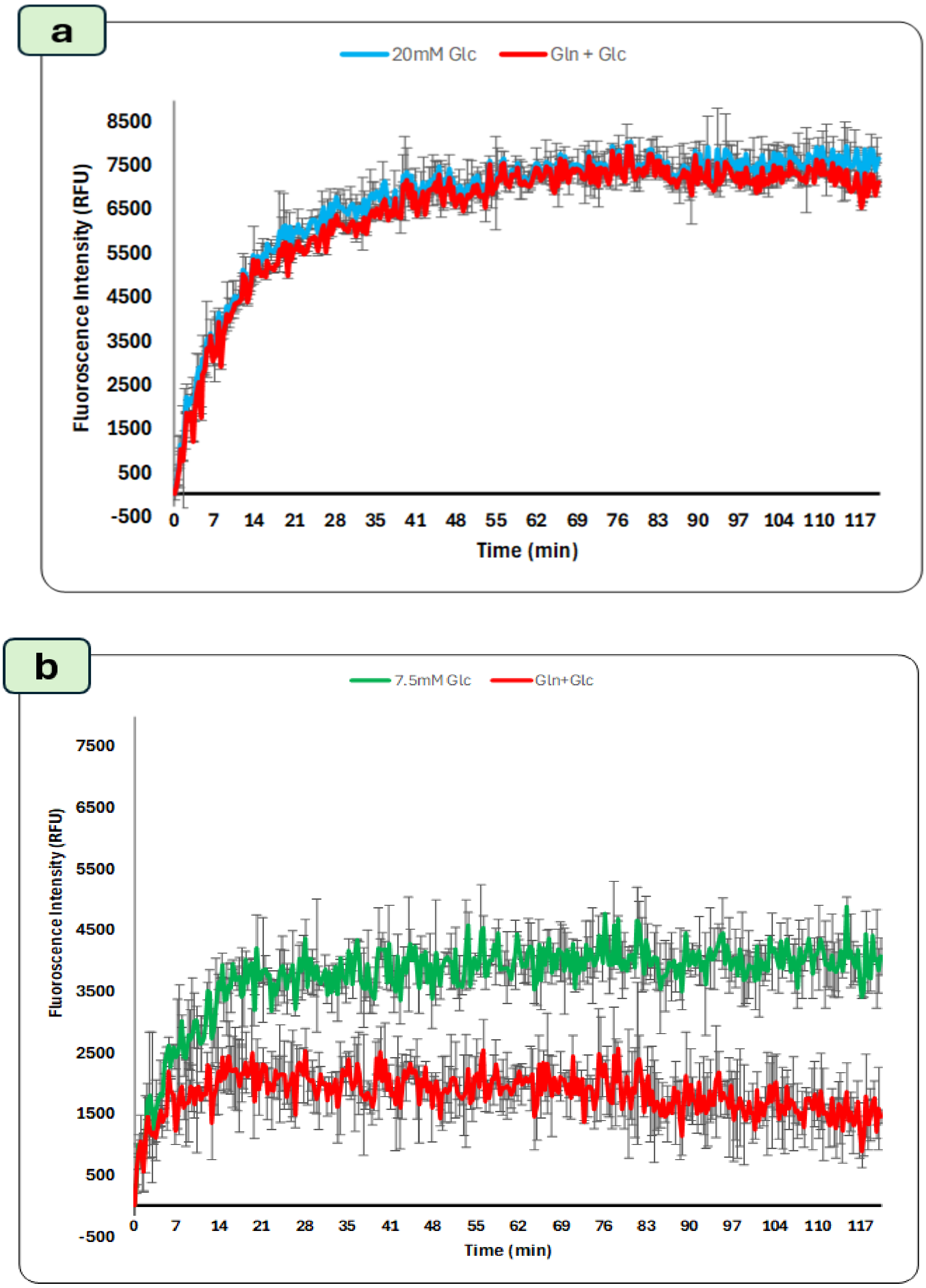

Figure 2S. Cellular responses to ECA kinetic changes with glucose alone (blue plot) versus a mixture of glucose and 2 mM glutamine (red plot). At a concentration of 20 mM glucose (a), no significant change is observed, while at a concentration of 7.5 mM glucose (b), a notable ECA change is observed upon glutamine addition. The error bars represent the standard deviation (STD) of triplicate measurements.

Table 1S: List of ordinary differential equations which are the basis of the model

No.	*ODE's*
1	d(Glc_ex)/dt = 1/media*(-(k_Glc*Glc_ex*(Capacity_Glc)))
2	d(Lac_ex)/dt = 1/media*((kf_3*Lac-kr_3*Lac_ex))
3	d(Gln_ex)/dt = 1/media*(-(k_Gln*Gln_ex*Capacity_Gln))
4	d(Glc)/dt = 1/cell*((k_Glc*Glc_ex*(Capacity_Glc)) - (kf_1*Glc*(1+Oligomycin*i2-[2DG]*i1)-kr_1*Pyr))
5	d(Pyr)/dt = 1/cell*((kf_1*Glc*(1+Oligomycin*i2-[2DG]*i1)-kr_1*Pyr) - (kf_2*Pyr-kr_2*Lac) - (kf_4*Pyr*(Capacity_M-TCA)-kr_4*TCA))
6	d(Lac)/dt = 1/cell*((kf_2*Pyr-kr_2*Lac) - (kf_3*Lac-kr_3*Lac_ex))
7	d(Gln)/dt = 1/cell*((k_Gln*Gln_ex*Capacity_Gln) - (kf_7*Gln))
8	d(TCA)/dt = 1/mitochondria*((kf_4*Pyr*(Capacity_M-TCA)-kr_4*TCA) - (kf_5*TCA*(1+Gln/10)*(1-Oligomycin*i2)) - (kf_6*TCA) + (kf_7*Gln))
9	d(ETC)/dt = 1/mitochondria*((kf_5*TCA*(1+Gln/10)*(1-Oligomycin*i2)))
10	d(CellComponents)/dt = 1/mitochondria*((kf_6*TCA))

Table 2S: Optimised values of rate constants (min^{-1}) in the kinetic model

Rate Constants (k)	Value
K_Glc	0.00012 *
kf_1	1 *
kr_1	0.01 *
kf_2	1 *
kr_2	0.1 *
kf_3	1 *
kr_3	0.01
kf_4	0.1 *
kr_4	1 *
kf_5	1 *
kf_6	1 *
kf_7	0.1
K_Gln	0.0001
i1	0.1 *
i2	0.1 *

Table 3S: Optimised values of initial condition parameters in the kinetic model

Initial Condition	Value
Glc_ex	7500
Gln_ex	2000
Capacity_Glc	200
Capacity_Gln	100
Capacity_M	50
